# Comparative Analysis of Viral Gene Expression Programs during Poxvirus Infection: A Transcriptional Map of the Vaccinia and Monkeypox Genomes

**DOI:** 10.1371/journal.pone.0002628

**Published:** 2008-07-09

**Authors:** Kathleen H. Rubins, Lisa E. Hensley, George W. Bell, Chunlin Wang, Elliot J. Lefkowitz, Patrick O. Brown, David A. Relman

**Affiliations:** 1 Department of Microbiology and Immunology, Stanford University School of Medicine, Stanford, California, United States of America; 2 Biochemistry, Stanford University School of Medicine, Stanford, California, United States of America; 3 Howard Hughes Medical Institute, Stanford University School of Medicine, Stanford, California, United States of America; 4 US Army Medical Research Institute of Infectious Diseases, Frederick, Maryland, United States of America; 5 Whitehead Institute for Biomedical Research, Cambridge, Massachusetts, United States of America; 6 University of Alabama at Birmingham, Birmingham, Alabama, United States of America; 7 Department of Medicine, Stanford University School of Medicine, and Veterans Affairs Palo Alto Health Care System, Palo Alto, California, United States of America; Columbia University, United States of America

## Abstract

**Background:**

Poxviruses engage in a complex and intricate dialogue with host cells as part of their strategy for replication. However, relatively little molecular detail is available with which to understand the mechanisms behind this dialogue.

**Methodology/Principal Findings:**

We designed a specialized microarray that contains probes specific to all predicted ORFs in the Monkeypox Zaire (MPXV) and Vaccinia Western Reserve (VACV) genomes, as well as >18,000 human genes, and used this tool to characterize MPXV and VACV gene expression responses *in vitro* during the course of primary infection of human monocytes, primary human fibroblasts and HeLa cells. The two viral transcriptomes show distinct features of temporal regulation and species-specific gene expression, and provide an early foundation for understanding global gene expression responses during poxvirus infection.

**Conclusions/Significance:**

The results provide a temporal map of the transcriptome of each virus during infection, enabling us to compare viral gene expression across species, and classify expression patterns of previously uncharacterized ORFs.

## Introduction

The family *Poxviridae* consists of large double-stranded DNA viruses, which replicate exclusively in the cytoplasm of cells. Members of the *orthopox* genus include variola, the causative agent of human smallpox, monkeypox (MPXV) and vaccinia (VACV).

Monkeypox (MPXV) infection causes severe disease in both humans and non-human primates, and is an emerging infectious disease, with cases observed in Africa [1]–[4], and recently, in the United States [5], [6].

The genomes of several MPXV strains have been sequenced [7]–[10], however virtually no modern molecular biology has been applied to the study of live MPXV virus. Much can be inferred about the life cycle, gene transcription, and putative host immune counter-defenses from the genome sequence of MPXV, and comparison to related poxviruses [7], [8]. However, none of these inferred features have been tested directly *in vitro* with MPXV virus. Moreover, the terminal ends of the genome, which encode immune-modulating and virulence genes, are the regions of the genome that vary the most between MPXV and VACV [8].

The mechanisms of VACV transcription are well described [11]. Vaccinia virus transcription proceeds in three phases. During the first, early transcriptional phase, factors are expressed that are involved in viral DNA synthesis, intermediate gene expression, and modulation of the host anti-viral response [11]. It is believed that approximately half of the vaccinia virus genome is transcribed during this phase, before DNA replication [12], [13]. The class of genes expressed during the second or intermediate phase, immediately after DNA replication, is a much smaller group [14], [15], mainly trans activating factors for late gene transcription. The third or late class of VACV genes encodes structural components of the virus, as well as components of the early transcriptional apparatus so that they can be synthesized and packaged for the next round of infection [11].

We examined the temporal features of *in vitro* infection with monkeypox and vaccinia in several different human cell types, and in this study, focused on patterns of viral gene expression. For this purpose, we developed a combination poxvirus-human DNA microarray. DNA microarray profiling has been applied successfully to the study of herpesvirus genomes (a similarly complex DNA virus) using both short [16] and long oligonucleotide arrays [17]–[19]. Our results provide a complete transcriptional map of the vaccinia and monkeypox genomes and clarify long-standing assumptions about the poxvirus life cycle in host cells.

## Results

In order to understand the dynamics of viral gene expression on a global scale, we performed high-resolution timecourse experiments with vaccinia (VACV) and monkeypox (MPXV) viruses. We infected primary human monocytes, primary human fibroblasts, and HeLa cells with Vaccinia WR or Monkeypox Zaire at a high multiplicity of infection (in order to maximize the proportion of infected cells and synchronize the infection), and mapped the transcriptional response of these viruses during one round of the infection cycle.

### Design and validation of a poxvirus-human DNA microarray

**Figure 1:**
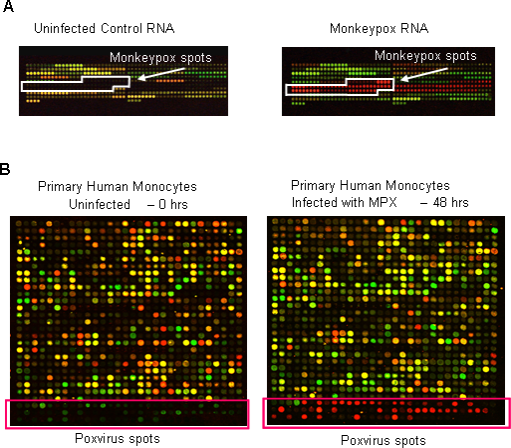
Validation of poxvirus-human microarray. (A) Monkeypox ORF PCR products were tested by printing the 384-well plate of monkeypox products on an array with three 384-well plates of human gene clones. Each spot from one well of a 384-well plate was printed 8 times (across, in rows). Two of the three 384-well plates of human clones were printed, then the monkeypox plate was printed (monkeypox spots start at the fifth row down, seventeenth spot from the left), and then the third 384-well plate of human clones was printed. Test hybridizations were performed using control uninfected cell line RNA from K562 cells (Uninfected Control RNA) or RNA from human PBMCs infected with monkeypox at an MOI of 1 at 24 hours post infection (Monkeypox RNA) labeled with Cy 5 versus common reference RNA (Stratagene, Inc.) labeled with Cy3. (B) 384-well plates of the ORF Monkeypox ORF PCR products were spotted in duplicate on the human array (bottom of the sector, Poxvirus spots). One of the 48 sectors is shown. Test hybridizations were performed with Cy5-labeled RNA from uninfected primary human monocytes or Cy5-labeled RNA from MPXV-infected primary human monocytes at 48 hours post-infection, versus a Cy 3 labeled common reference pool (Universal Human Reference, Stratagene Inc.) combined with an equal mix of poxvirus transcripts from all timepoints.

Because our overall goal was to monitor both viral and host gene expression simultaneously during the course of infection, we developed and tested a specialized poxvirus-human DNA microarray. Using the monkeypox [7], [8] and vaccinia [20] genomes, and software developed in our lab [21], we designed primers for all 190 predicted open reading frames (ORFs) in the MPXV-ZAI genome with an overall PCR success rate of 94.7% (180/190 ORFs), and all 217 predicted open reading frames (ORFs) in the vaccinia-WR genome with an overall PCR success rate of 94.9% (206/217 ORFs). In a preliminary set of experiments, these MPXV and VACV DNAs were printed on a microarray along with 1152 human cDNAs as controls. Test hybridizations were performed using control uninfected human K562 cell line RNA and RNA from fresh human PBMCs infected with MPXV-ZAI (24 hrs post-infection; MOI = 1) (Figure 1A). The MPXV array elements hybridized specifically to RNA from the infected sample (red MPXV spots, Figure 1A). Those same spots appeared black (no hybridization) when RNA from uninfected control cells was used (Figure 1A). The mean pixel intensity of the MPXV array elements in the infected sample was 1,333.63, which was significantly higher than the mean pixel intensity of the corresponding elements in the uninfected control hybridization (219.52). In addition, we also tested the full poxvirus-human microarray using RNA samples from an *in vitro* infection with monkeypox. Figure 1B shows results using primary human monocytes, either uninfected or 48 hrs post infection in the red channel, with a reference consisting of a mix of poxvirus transcripts and human transcripts in the green channel. Thus, the poxvirus-human arrays were able to capture nearly all poxvirus transcripts with little background hybridization from the uninfected control. Using two different types of human primary cells, we were able to detect transcripts representing 98.16% and 95.58% of poxviral genes (as defined by signal >2.5 fold over background at one of the timepoints) in VACV-WR-infected fibroblasts and monocytes, respectively, and 99.02% and 98.65% of poxvirus genes in MPXV-ZAI-infected fibroblasts and monocytes, respectively.

### Temporal transcription program of poxviral genes

**Figure 2:**
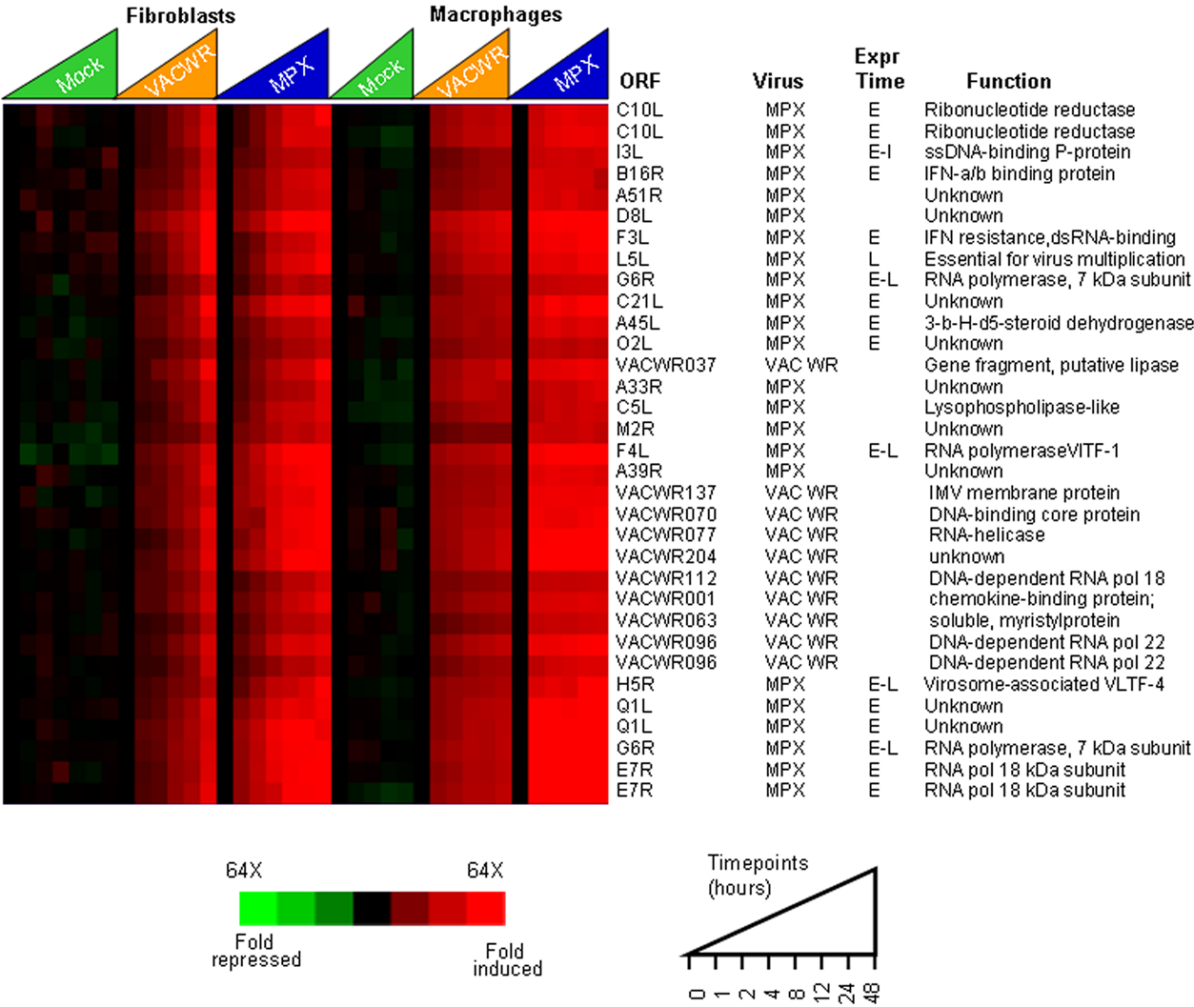
Early viral gene expression during poxvirus infection. A subset of the complete monkeypox and vaccinia transcriptome representative of the early transcripts. Timecourses are as follows: Mock = mock infection (media only), VACV-WR = Vaccinia-Western Reserve, MPXV = Monkeypox-Zaire. Open Reading Frame (ORF), virus species, predicted timing of expression from VACV (Expr Time) [8] and protein function are displayed. Poxvirus ORFs were spotted in duplicate on arrays, and resulting gene expression values relative to baseline (time 0) are displayed. The intensity of the color reflects the magnitude of the change from baseline (−64 fold to 64 fold change from baseline).

**Figure 3:**
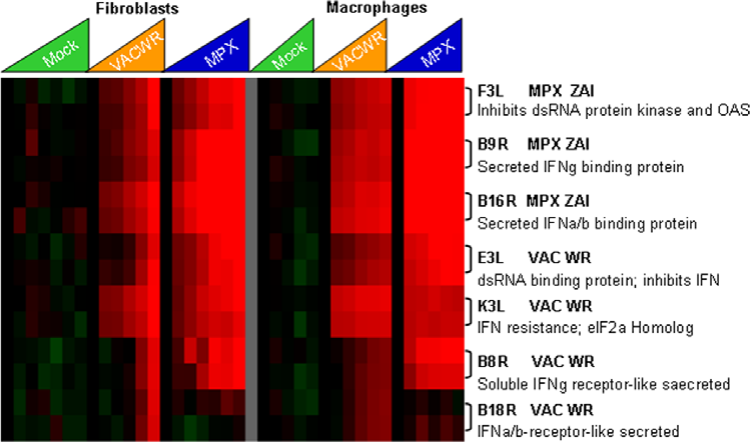
Detection of poxvirus gene transcripts-interferon modulators. Poxvirus genes whose products are known or predicted to modulate interferon signaling. Timecourses are as follows: Mock = mock infection (media only), VACV-WR = Vaccinia-Western Reserve, MPXV = Monkeypox-Zaire. The features of this display are similar to those in Figure 2.

As a means of exploring global patterns of poxvirus gene expression, we developed a map of the MPXV and VACV transcriptomes in human primary monocytes and fibroblasts using the poxvirus-human arrays and samples taken at 0, 1, 2, 4, 6, 8, 12, and 24 hours post infection. We identified temporal expression classes of poxvirus genes during infection, including viral transcripts that were induced early (1–2 hrs post infection), and transcripts that were induced late in infection (>4 hrs post infection) (Figs 2 and 3).

171 MPXV and VACV ORFs were expressed early (subset shown in Figure 2), including genes whose products are known to be involved in viral DNA replication (DNA polymerase VACVWR065, viral DNA processivity factor VACVWR141, uracil DNA glycosylase MPXV E4R), RNA transcription (DNA-dependent RNA polymerase subunits VACVWR112, VACVWR 124, VACVWR 144 and VACVWR 096 and MPXV G6R, F4L, L4R), and host immune modulating factors (secreted Il-18 binding protein VACVWR013, Toll/IL1-recptor suppressor VACVWR172, secreted TNFa binding protein MPXV J2L, 3-β-hydroxy-δ5-steroid dehydrogenase MPXV A45L, and secreted EGF-like growth factor MPXV D3R). Being that poxvirus immune modulators are important for pathogenesis and viral interaction with the host cell environment, we used the poxvirus-human microarray to determine if, and when the putative MPXV interferon-modulating proteins were expressed during infection. These poxvirus transcripts were detectable in both MPXV and VACV infection as early as 2 hours post-infection, and reached levels as high as 138-fold by 4–8 hours post infection (Figure 3). In addition to the known early and immunomodulatory genes, 50 ORFs (from MPXV or VACV) in the early expression cluster encode products with unknown function.

**Figure 4:**
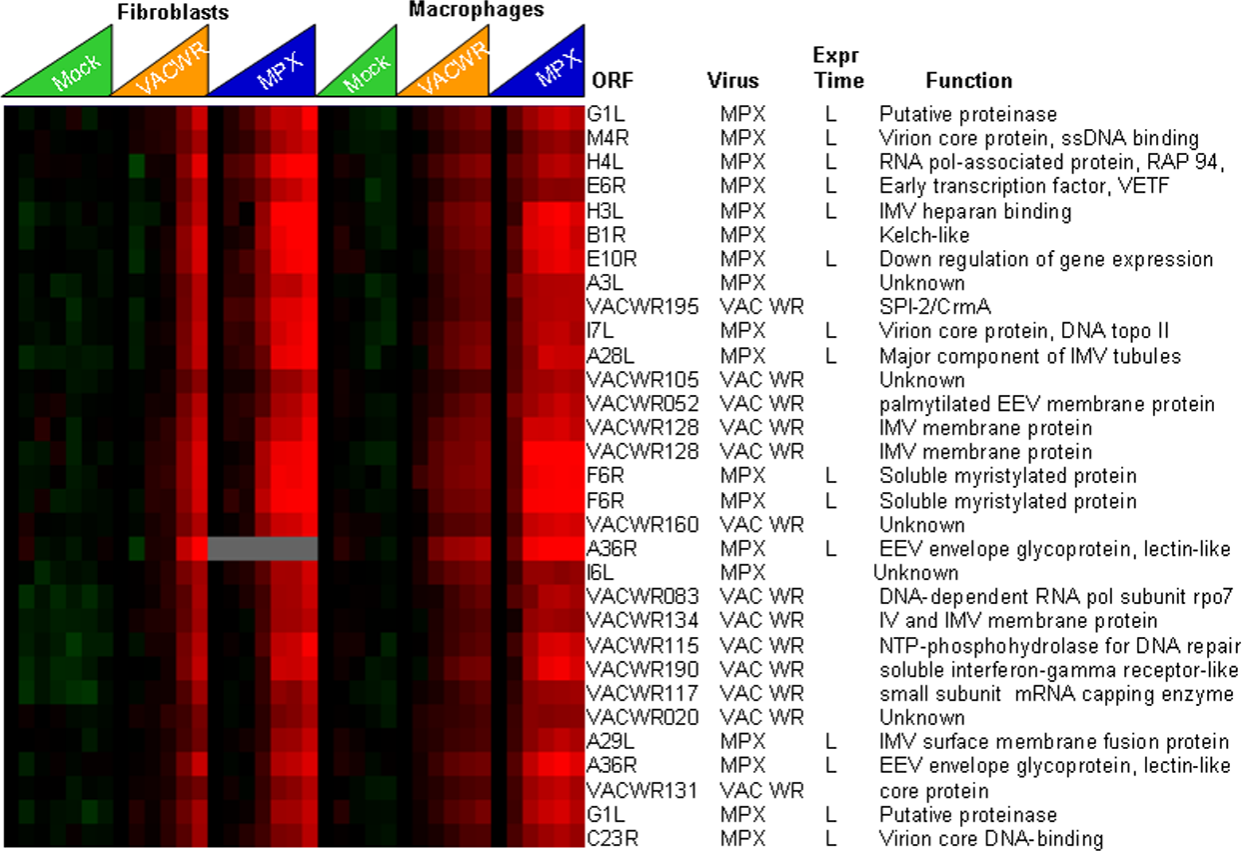
Late viral gene expression during poxvirus infection. A subset of the complete monkeypox and vaccinia transcriptome representative of the late transcripts. Timecourses are as follows: Mock = mock infection (media only), VACV-WR = Vaccinia-Western Reserve, MPXV = Monkeypox-Zaire. The features of this display are similar to those in Figure 2.

The genes expressed at late timepoints (subset shown in Figure 4) comprised 240 ORFs, including those encoding structural proteins and major virion components (major virion core protein 4a VACVWR129, A11L and 4b VACVWR122, MPXV A4L, IMV membrane protein A28L, major component of IMV tubules MPXV A28L, virion core DNA-binding MPXV C23R), and early transcription factors that are packaged into the virion (large and small subunits of viral early transcription factor VETF, VACVWR126, VACVWR111, MPXV A8 and MPXV E6R, and RAP94 required for transcription of early promoter templates VACVWR102). 60 MPXV or VACV ORFs in this cluster encode products with no known function.

**Figure 5:**
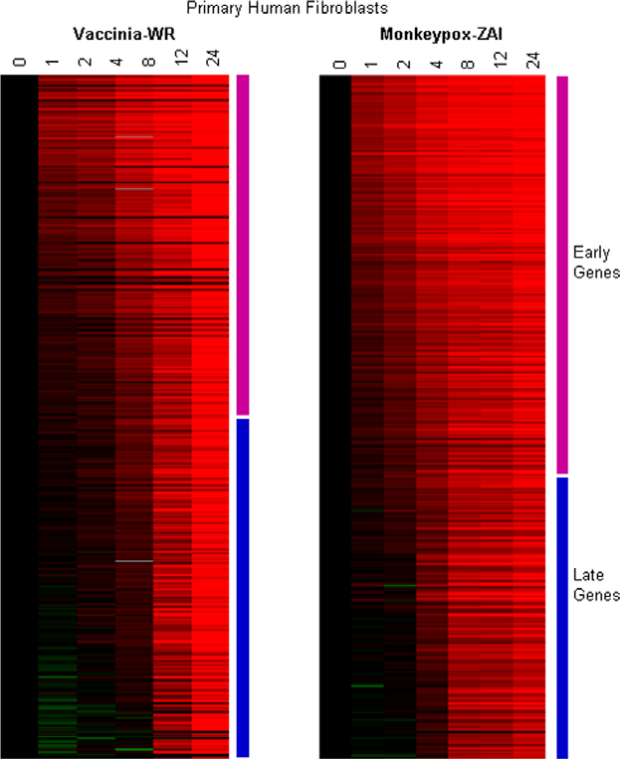
Self Organizing Maps-temporal transcription program. Self Organizing Maps (SOMs, 10 nodes, 100,000 gene iterations) of the gene expression values relative to baseline (time 0) for VACV ORFs during VACV WR infection and MPXV ORFs during MPXV infection of primary human fibroblasts. The intensity of the color reflects the magnitude of the change from baseline (−64 fold to 64 fold change from baseline).

As an alternative approach, we organized the transcriptome of each virus in each cell type into a temporal map based on time of transcription initiation, using Self Organizing Maps (SOMs) [22], [23] (10 nodes, 100,000 gene iterations). SOMs of the VACV ORFs only during VACV WR infection and of the MPXV ORFs only during MPXV infection (of primary human fibroblasts) are shown in Figure 5. The overall temporal programs were similar between the two viruses, with approximately half the viral genes expressed at early timepoints (1–2 hours post infection), and half expressed at late timepoints (4–24 hours post infection. The early transcripts appeared to remain at steady state levels during the late time period. In addition, transcripts representing almost all viral genes appear to be detected at 24 hours post infection.

### Viral species-specific transcripts

**Figure 6:**
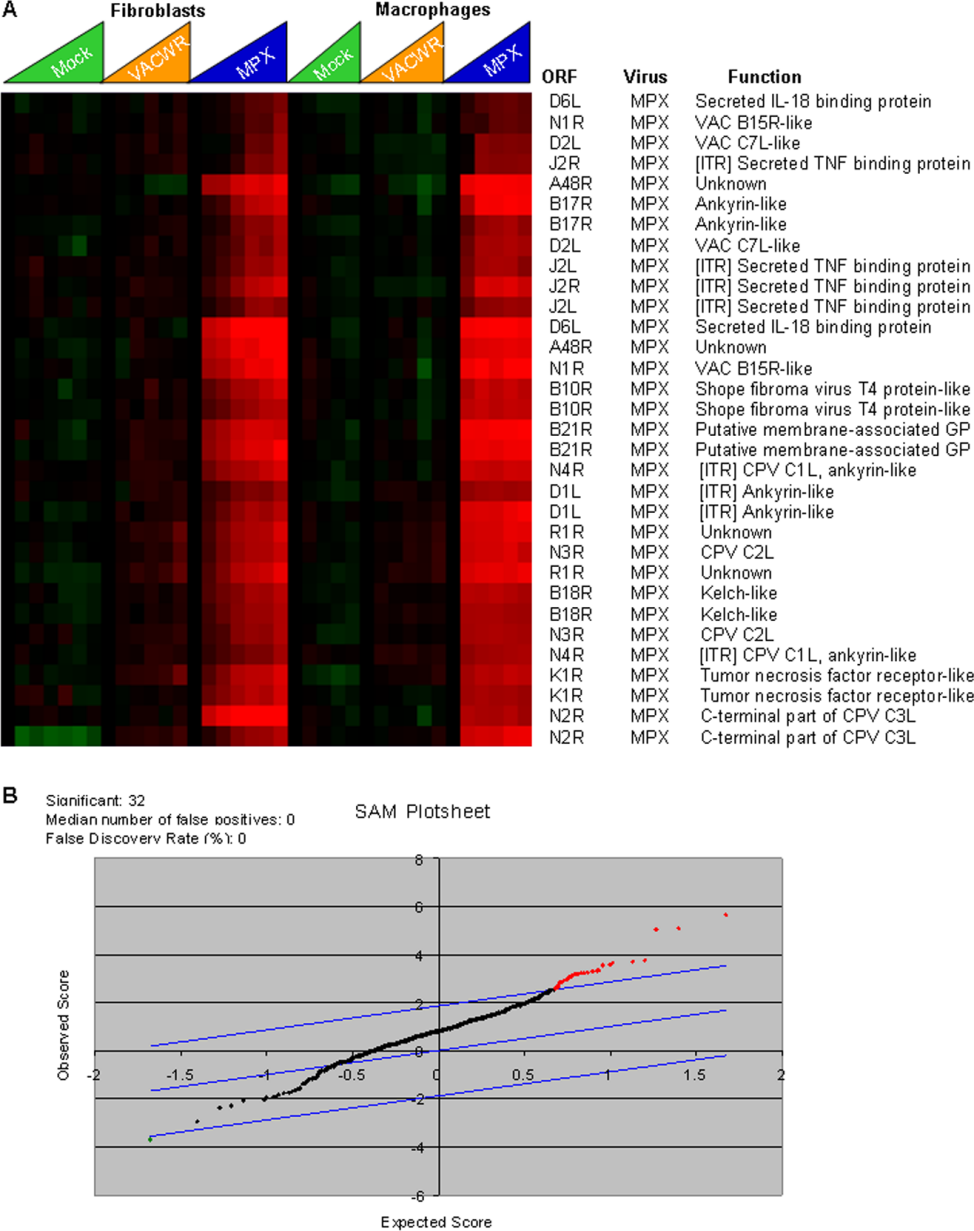
Virus species-specific transcripts. (A) MPXV-specific transcript cluster (17 ORFs) was selected from overall unsupervised hierarchical cluster of 407 MPXV and VACV genes (data not shown) and the resulting gene expression values relative to baseline (time 0) are shown. Mock = mock infection (media only), VACV-WR = Vaccinia-Western Reserve, MPXV = Monkeypox-Zaire. The features of this display are similar to those in Figure 2. (B) Significance Analysis of Microarrays (SAM) plot of 22 significant genes differentially expressed in VACV vs MPXV, (two class paired timecourse, 1000 iterations, delta of 1.88, median false discovery rate = 0).

Several clusters of genes in the transcriptome map showed expression by one virus, but not the other (e.g., MPXV-specific cluster, Figure 6A). 16 MPXV ORFs and 12 VACV ORFs showed strong expression by the corresponding virus, but little or no expression by the other virus. 17 of the 28 genes detected as virus species-specific (i.e. expression detected by MPXV spots but not VACV spots or vice versa) were significantly differentially expressed by VACV or MPXV, based on Significance Analysis of Microarrays (SAM) [24] (two class paired timecourse, 1000 iterations, delta of 1.88, median false discovery rate = 0), regardless of host cell type (Figure 6B). We aligned the MPXV-ZAI and VACV-WR genomes using Base-by-Base [25] and determined that the array would detect ORFs as species specific with as little as 12.34% difference at the nucleotide level.

### Mapping the virus transcriptome onto the physical map of the viral genome

**Figure 7:**
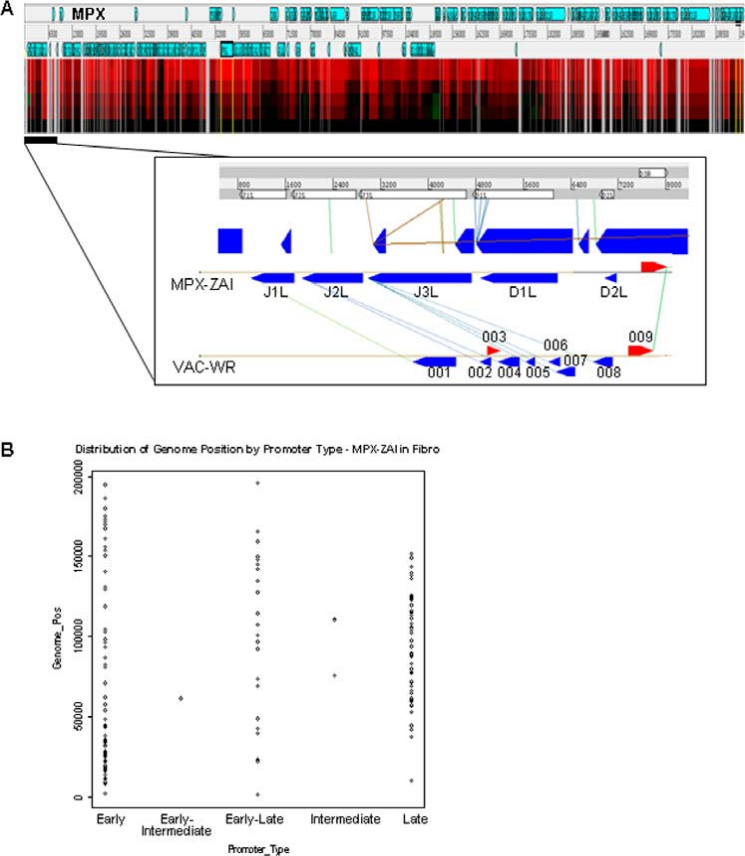
Mapping of virus transcriptome to genome and distribution of promoters. (A) Top panel: Overview alignment of expression data for all MPXV ORFs according to genome position, bottom panel: a zoomed-in view of the left terminal region, MPXV ZAI and VACV WR genome alignment and homology of viral gene families. The MPXV ZAI and VACV WR genomes were then aligned using the viral orthologous cluster software package (VOCS) [25], [41], and the transcriptome expression data were mapped onto the alignment. Genes predicted to be expressed in MPXV [8] had corresponding hybridization data (MPXV J1L, J2L, J3L, D1L, D2L and D3R and VACVWR 001, 003 and 009), while ORFs that are predicted to be fragmented or missing in VACV (the ORFs corresponding with the MPXV J3L, D1L and D2L) were not detected with the MPXV probes. (B) Distribution of genome position start sites by promoter type for MPXV-ZAI ORFs in fibroblasts.

We aligned the expression data for all virus ORFs according to genome position. The condensed overview is shown in Figure 7A (top panel); a zoomed-in view of the left terminal region is shown in the bottom panel. Overall, the transcriptional timing did not appear to have a systematic relationship to position in the genome, with the exception of early transcripts, which were disproportionately distributed towards the termini of the genome, and late transcripts which were concentrated in the central regions of the genome (p<0.001 by t-test, Figure 7B). MPXV ORFs whose predicted orthologs are fragmented in VACVV-WR showed MPXV species-specific expression, as described above.

### Further delineation of early and late viral gene expression

**Figure 8:**
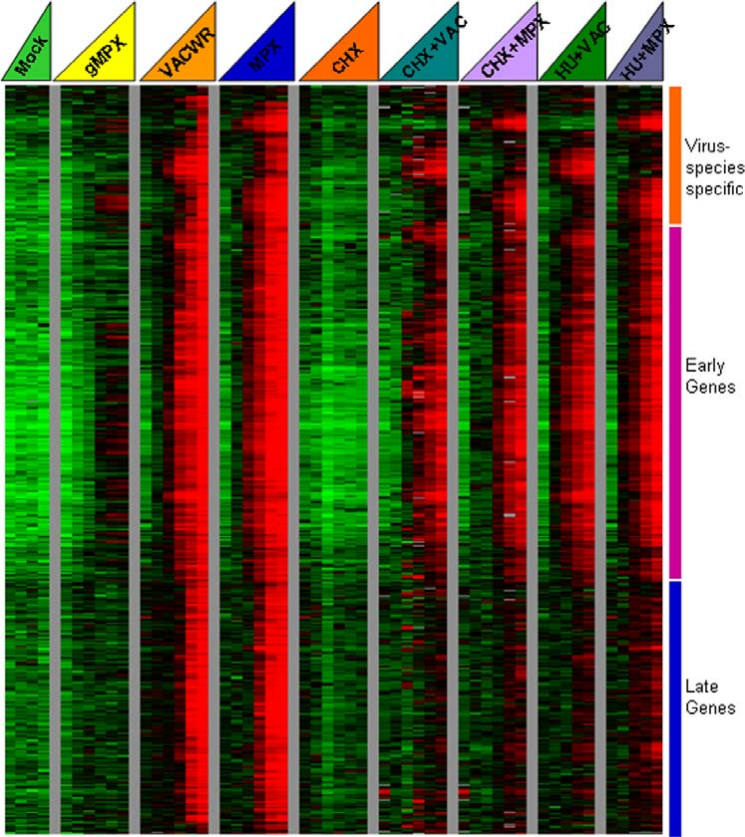
Block of late virus transcription with cycloheximide and/or hydroxyurea. Hierarchical clustering of 814 poxvirus elements. The level of expression of each gene, in each sample, relative to the mean expression level of that gene across all of the samples was determined. The intensity of the color reflects the magnitude of the change relative to the rest of the samples (−64 fold to 64 fold change). Mock = mock infection (media only); γ-MPXV = killed (gamma-irradiated) Monkeypox-Zaire; VACV-WR = Vaccinia-Western Reserve; MPXV = Monkeypox-Zaire; CHX = cycloheximide; CHX+VACV = VACV infected, CHX added immediately post-infection; CHX+MPXV = MPXV infected, CHX added immediately post-infection; HU+VACV = VACV infected, hydroxyurea (HU) added immediately post-infection then washed out at 2 hours post-infection and CHX added; HU+MPXV = MPXV infected, HU added immediately post-infection then washed out at 2 hours post-infection and CHX added.

In order to identify early and late expressed poxvirus genes more accurately, we performed infection timecourses in primary fibroblasts and in HeLa cells, according to the conditions used by Baldick et al. [15]. In parallel, we mock infected cells, and infected cells with killed MPXV, VACV-WR or MPXV-ZAI. Parallel infections with VACV and MPXV were also performed in which cycloheximide (CHX) was added to the media immediately after viral adsorption, so as to block protein synthesis (including synthesis of transcription factors necessary for intermediate and late virus gene expression) and allow only expression of early poxvirus ORFs. In another parallel set of experiments, we infected with VACV or MPXV, then added hydroxyurea (HU) to the media immediately after viral adsorption, so as to block viral DNA replication and allow early viral proteins to accumulate; we then washed out HU at 2 hours post-infection and added CHX to allow transcription of intermediate but not late virus genes. The resulting gene expression data are shown in Figure 8. CHX alone and HU plus CHX efficiently blocked transcription of the viral genes that we had previously classified as late-expressed genes, for both MPXV and VACV (blue bar, 3^rd^ and 4^th^ triangle, versus 6^th^–9^th^ triangle, Figure 8). We developed a model to distinguish between early (pre-genome replication) and late (post-genome replication) transcription, based on expression value at 12 hours post MPXV or VACV infection in untreated versus CHX or CHX+HU-treated cells (Supplementary Table 3). This model agreed with bioinformatics-based promoter predictions for vaccinia late genes (http://www.poxvirus.org/vaccinia_orthologs.asp)for 65 out of 94 vaccinia genes with a predicted late promoter (P<0.001 by exact binomial test). However it only agreed with predicted promoters for 27 of 64 genes with predicted early promoters, perhaps due to the number of genes with predicted dual early and late promoters. This model allows for classification of genes with no previous information as pre-DNA replication (early) or post-DNA replication (late), and may aid in studies on poxviral genes with previously unknown function.

## Discussion

In order to examine the global temporal program of poxvirus transcription during a synchronous infection, we developed a specialized poxvirus-human microarray, containing probes representing every annotated ORF in the MPXV and VACV genomes. We used this array to map the complete MPXV and VACV transcriptomes with relatively dense temporal sampling. We categorized all genes in the MPXV and VACV genomes according to their transcriptional temporal profile (Figs 5 and 8). These experiments allowed us to classify genes into the general categories of “early” or “late” based on timing of hybridization signal and weather or not viral DNA replication was required for transcript detection. We observed the expected functional categories of genes in each temporal class (i.e., genes involved in viral transcription and DNA replication, as well as host immune modulation were expressed early, and structural proteins, major virion components and factors needed for early transcription in the next cycle of replication were expressed late). However within each class, there was variation as to the exact timing of transcription.

The standard vaccinia classification of genes as early, intermediate, or late is based on temporality of transcription initiation. However this study is designed to detect the timing of the presence of transcripts, as assessed by hybridization. The methods utilized in this work are able to predict genes transcribed early or late in the replication cycle, but have more difficulty distinguishing early-only versus genes with an early and late promoter since early-only transcripts may persist and be detected at late times. Some genes have both an early and late promoter (as demonstrated experimentally as well as predicted with bioinformatics methods). These genes will have an expression pattern that behaves as an early gene during the initial part of the life cycle, but as a late gene during the latter part, potentially leading to partial, but not complete blockage of transcript detection when viral DNA replication is blocked. While we have not attempted to clearly identify genes expressed both early and late in the replication cycle, these genes would presumably comprise a subset of the early expressed genes identified in this study.

We aligned the expression data for all virus ORFs according to genome position. There did not appear to be a consistent relationship between transcriptional timing and specific regions of genome organization/structure, although there was a general tendency for early transcripts to be located towards the termini and late transcripts towards the central regions of the genome.

Many of the vaccinia genes have been temporally categorized and the promoter sequences for early and late promoters have been described [26]–[28]. Yet, 34% of predicted VACV-WR genes do not have a reported expression phase, and 52 out of 218 VACV-WR genes are of unknown function [29], [30]. In addition, aside from bioinformatics predictions from the MPXV genome sequence, nothing is known about the expression or timing of any MPXV transcripts. With this complete transcriptome map of both VACV and MPXV, many genes with previously unknown function can now be classified as early, or late, thereby providing clues to their function. Our poxvirus-human microarray was able to detect the viral transcripts encoding the predicted MPXV interferon-modulating genes. These results provide some of the first evidence of specific MPXV immunomodulators being expressed during live viral infection.

We noted that in the SOM analysis of the transcriptome, the entire set of viral ORFs appeared to be transcribed by 24 hours (Figure 5). While poxvirus early transcripts have a defined termination signal, late transcripts have heterogeneous 3′ ends as the early termination signal is not recognized by the viral late transcriptional apparatus. This “run-through transcription” presents the possibility of multi-ORF transcripts, where the transcript of a late gene continues into the next downstream open reading frame, due to the close proximity of poxvirus ORFs and relatively small intergenic spacing. Such multi-ORF transcripts would hybridize to array elements representing each of the included genes. To address this issue, we used cycloheximide and hydroxyurea treatments to identify genes whose expression pattern depended on viral DNA replication and protein synthesis and thus more clearly segregate early and late classes of viral genes. The resulting data clearly separated the transcriptional profiles into early and late categories (Figure 8), and demonstrated that signal intensities of early genes at later timepoints were not due to late run-through transcription.

Microarrays developed for both Herpes Simplex Virus (HSV)-1 [17] and HSV-2 [18] have enabled transcriptional profiling of each virus individually, as well as multi-species analysis of herpesvirus transcriptomes. While a similar overall categorization of HSV-1 and 2 kinetic classes was observed for orthologs, comparative analysis demonstrated differential patterns of four HSV transcripts which play a functional role in the nuclear organization and localization of viral DNA [19] using cross hybridization of HSV-2 transcripts on HSV-1 probes. For poxviruses, we have observed a similar cross-hybridization between VACV and MPXV array probes with the opposite viral transcript (Figures 2, 4, and 6). We were able to identify 16 MPXV ORFs and 12 VACV ORFs which are differentially expressed, with 17 of the 28 virus-species specific genes statistically significantly by Significance Analysis of Microarrays (False discovery rate = 0), regardless of host cell type. This microarray was not designed to be diagnostic; by using whole-genome arrays for each virus, probes were designed independent of degree of cross-hybridization. However the species specificity profiling analysis allows us to discriminate species-specific virus gene expression with little as 12.34% difference at the nucleotide level. Many of the species-specific genes are located in the highly variable terminal genomic regions and in the inverted terminal repeats. These may be ORFs which are significantly divergent at the nucleotide level as to not cross-hybridize to the corresponding ortholog, as well as viral ORFs which have been fragmented and are not expressed at all in the corresponding viral species. These differentially expressed ORFs include transcripts for MPXV proteins which have not previously been characterized as well as ORFs which are critical to modulation of host defenses (viral suppressors of Toll-like receptor signaling, secreted TNFα and IFNα/β decoy receptor, and IL-18 binding protein). These differentially expressed genes may be critical factors in elucidating pathogenesis differences between MPXV and VACV.

During review of our paper, a study was published examining VACV transcriptional responses in HeLa cells using short-oligonucleotide microarray profiling [31]. Overall, transcription patters were observed to be similar between the short-oligonuceotide and cDNA microarray platforms. Our analysis extends these results to MPXV, as well as primary cell culture, which may be a more relevant biological system. This study represents the first analysis of the complete virus transcriptome for both VACV and MPXV. This map of the transcriptional timing of all viral genes can be used to interrogate functions of unknown viral genes and dissect the intricate dialogue between virus and host.

## Materials and Methods

### Cells, and culture conditions

In order to obtain primary human monocytes, elutriated monocytes were collected from two different healthy donors through the NIH Blood Bank and also from Clonetics (San Diego, CA). The cells were isolated according to conventional procedures and cultured in 6- or 12-well plates containing RPMI 1640 medium and 10% heat-inactivated fetal calf serum (FCS, Invitrogen, Carlsbad, CA). The use of primary human cells for research purposes was approved by the Institutional Review Board of Stanford University. Monocytes were cultured for 2–6 days to allow differentiation into monocyte-like cells. Primary human dermal fibroblasts were either derived from skin samples obtained at autopsy, after removal of keratinocytes and endothelial cells, as described [32] (S100), or obtained from Clonetics (NHDF). S100 primary fibroblasts were cultured in Dulbecco's Modified Eagle's Medium (DMEM) with 10% FCS, glutamine, and 100 units penicillin-streptomycin (Invitrogen, Carlsbad, CA); NHDF primary fibroblasts were cultured in fibroblast growth medum 2 (Clonetics, San Diego, CA) supplemented with 2% FCS human fibroblast growth factor-B, insulin, and gentamicin/amphotericin-B, according to the manufacturer's directions. HeLa cells (ATCC, Manassas, VA) were cultured in DMEM with 10% FCS, glutamine, and 100 units penicillin-streptomycin.

### Viral infection

Cells were plated in 6- or 12-well plates and rested for 24 hours. Cells were infected at a multiplicity of infection (MOI) of 10 with either Vaccinia New York Board of Health (Wyeth, Dryvax) (VACV-NYCBH), Vaccinia Western Reserve (VACV-WR, [33], kindly provided by B. Moss), or Monkeypox-Zaire (MPXV-ZAI, CDC isolate V79-I-005 [7]). Control live virus infections were performed at an MOI of 10 with Ebola-Zaire (EBOV-ZAI) [34]. Control killed virus infections were performed with gamma-irradiated MPXV-ZAI. For preparation of killed MPXV, the stock of MPXV-ZAI was split into equal aliquots, half was used for the live virus infection, and half was subjected to 1×10^6^ rads of gamma-irradiation from a ^60^Cobalt source. Mock infections were performed using culture medium free of any viruses. All viral infections were performed at U.S. Army Medical Research Institute of Infectious Diseases, Frederick, MD at Biosafety Level 3 (MPXV) or 4 (EBOV-ZAI) in accordance with NIH/CDC Biosafety in Microbiological and Biomedical Laboratories guidelines, as well as in accordance with CDC Select Agent regulations.

After incubation of virus with host cells for 1 hour at 37°C with periodic rocking, the virus-containing medium (or mock medium) was removed, cells were washed twice with phosphate-buffered saline, and replaced with fresh culture medium. Cells were then incubated for various times at 37°C. Samples were collected in duplicate or triplicate immediately prior to infection (“time zero”) for all timecourses in order to ensure a robust baseline for data analysis. The range of timepoints was chosen to cover to the first 24 hours post-infection, so as to include the expected periods of vaccinia early stage (1–2 hr [15]), intermediate stage (100 min–2 hr [15]), and late stage (140 min–24 hr [35]) transcription.

In some experiments, exogenous compounds were added to the media after infection to elicit specific responses. To examine the effects of blocking late virus transcription, HeLa cells were either mock infected (as described above), or infected with MPXV-ZAI at an MOI of 10. After unadsorbed virus was removed, fresh media containing 100 ug/mL (final concentration) of cycloheximide (CHX, Sigma, St. Louis, MO) was added to the mock- or MPXV-infected cells. In some experiments, hydroxyurea (HU, Sigma, St. Louis, MO) was added at a final concentration of 10 mM, instead of cycloheximide. HU was washed out at 2 hrs post infection, then fresh media containing 100 ug/mL final concentration of CHX was added and samples were collected at various timepoints. Cell cultures were visualized using phase-contrast microscopy at each timepoint and images captured.

### Plaque assays

Cell supernatants were collected for virus titration and soluble factor assays at the same times that RNA was harvested; the supernatant was frozen at −80°C until subsequent analysis. Supernatants were diluted from 10^−1^ to 10^−6^ in 200 uL of MEM. Diluted supernatants were added in duplicate to confluent monolayer cultures of Vero E6 cells and incubated for 1 hour at 37°C with periodic rocking. Supernatants were removed and a 0.5% agarose solution with 2% EBME, HEPES and 10% FBS was overlayed. Cells were incubated at 37°C for 4–5 days, followed by removal of the agarose overlay. Plaques were stained with 2 mL of crystal violet solution and counted.

### RNA preparation for microarray analysis

Total RNA from cells was harvested at each timepoint by removal of media and addition of 1 mL of TriPure (Roche Applied Science, Indianapolis, IN) directly to the infected/treated cells in the culture well. RNA was extracted using TriPure reagent and linearly amplified (Ambion MessageAmp, Ambion, Austin, TX) according to the manufacturer's instructions.

### DNA microarrays and hybridization

We used custom-designed DNA microarrays containing elements for both human and poxvirus genes. These microarrays contained 37,632 elements that represent approximately 18,000 unique human genes ([36], [37]). We designed primers for all 190 predicted open reading frames (ORFs) in the MPXV-ZAI genome (Genbank accession AF380138) and all 217 predicted ORFs in the vaccinia-WR genome (Genbank accession AY243312) [21]. Using purified MPZ-ZAI and VACV-WR virus genomic DNA as a template, PCR reactions were performed and PCR products were purified (according to the methods described at (http://www.microarray.org/doc/protocol/PCRprotocol.rtf) and each product was transferred twice to a 384-well plate (http://www.microarray.org/doc/protocol/96_to_384_well_transfer.rtf). Therefore, each PCR product representing a MPXV or VACV ORF was duplicated in the 384-well plate and was represented twice on the microarray. These poxvirus PCR products ranged in size from 100 to 348 bp (179 mean), see Supplementary Tables 1 and 2 for primer sequences and amplicons.

Fluorescently-labeled cDNA prepared from amplified RNA was hybridized to the array in a two color comparative format [36], [38], with the experimental samples labeled with one fluorophore (Cy-5) and a reference pool of mRNA labeled with a second fluorophore (Cy-3). The reference pool (Universal Human Reference, Stratagene Inc.) and a mix of poxvirus transcripts from all timepoints provided an internal standard to enable reliable comparison of relative transcript levels in multiple samples [36], [37], [39]. Fluorescent images of hybridized microarrays were acquired using the GenePix 4000B and GenePix 4200AL microarray scanners (Axon Instruments, Union City, CA).

The data discussed in this publication have been deposited in NCBIs Gene Expression Omnibus (GEO, http://www.ncbi.nlm.nih.gov/geo/) and are accessible through GEO Series accession number GSE11234. In addition array image files, raw and filtered data are available through the Stanford Microarray database http://smd-www.stanford.edu//

### Data Filtering and Analysis

Microarray images were analyzed with GenePix Pro 5.0 (http://www.axon.com/GN_GenePixSoftware.html, Axon Instruments, Union City, CA) and Spot Reader (Niles Scientific, Portola Valley, CA). Single spots or areas of the array with obvious blemishes were flagged and excluded from subsequent analyses. Fluorescence ratios (along with numerous quality control parameters; see GenePix Pro and SpotReader manuals) were stored in a custom database [40]. Clones that consistently behaved poorly (e.g., flagged data in >20% of arrays) were identified and excluded from all analyses. Fluorescence ratios were calibrated independently for each array by applying a single scaling factor to all fluorescent ratios from each; this scaling factor was computed so that the median fluorescence ratio of well-measured spots on each array was 1.0. All non-flagged array elements for which the fluorescent intensity in each channel was greater than 2.5 times the local background and for which the regression correlation of the pixels in each channel was at least 0.6 on at least 80% of arrays, were considered well-measured. Data were expressed as the log_2_ ratio of fluorescence intensities of the sample and the reference, for each element on the array [36], [38]. The data from multiple independent Time 0 samples were averaged to provide a robust baseline. The timecourse data were then zero-transformed (the value for each element at day 0 for a given time course was subtracted from the rest of the values for that element within that time course). The subset of elements that varied from the baseline by at least 3 fold in at least 3 samples was selected for further analysis. The data were hierarchically clustered using the Cluster program [23] and displayed using TreeView (http://rana.lbl.gov/EisenSoftware.htm).

### Classification of early vs. late genes from expression profiles

Data from 12-hours post-VACV or MPXV infection were normalized by scaling the mean of all probes to the global mean of each of these hybridizations. Probes from hybridizations of cells 12 hours post VACV or MPXV infection treated with CHX or HU+CHX were also scaled similarly across each group. To discriminate between early and late genes, a “lateness score” was calculated by subtracting the mean expression value of probes for cells infected with VACV or MPXV and treated by CHX or HU+CHX (n = 4) from that of cells infected with VACV or MPXV only (n = 2) and dividing by the standard deviation of the treated cells. Genes were ranked by this lateness score, with the top half (exhibiting the most reproducible effect of CHX or HU+CHX treatment; lateness score >1.6) being assigned a “late” mode of expression regulation and the remaining genes assigned an “early” mode (Supplementary Table 3).
